# Chick chorioallantoic membrane model as a preclinical platform for cryoablation studies

**DOI:** 10.1186/s41747-025-00592-z

**Published:** 2025-05-27

**Authors:** Michael Scheschenja, Jarmila Jedelská, Eva Juchems, Marc Weinmann, Axel Pagenstecher, Frederik Helmprobst, Malte Buchholz, Marina Tatura, Jens Schaefer, Udo Bakowsky, Alexander M. König, Andreas H. Mahnken

**Affiliations:** 1 Department of Diagnostic and Interventional Radiology, University Hospital Marburg, Philipps-University Marburg, Marburg, Germany; 2 Mouse Pathology and Electron Microscopy—Core Facility, Institute of Neuropathology, Philipps-University Marburg, Marburg, Germany; 3 Department of Gastroenterology, Endocrinology, Metabolism and Infectiology, Philipps-University Marburg, Marburg, Germany; 4 Department of Pharmaceutics and Biopharmaceutics, Philipps-University Marburg, Marburg, Germany

**Keywords:** Chick embryo, Chorioallantoic membrane, Cryoablation, Radiology (interventional), Xenografts

## Abstract

**Background:**

The chick chorioallantoic membrane (CAM) model has been utilized for radiofrequency ablation and electroporation, but not yet for cryoablation. This study aims to evaluate the feasibility of the CAM model for preclinical cryoablation research.

**Methods:**

Two cryoablation protocols were established for the study: 120 s-freeze-120 s-thaw-120 s freeze (120 s protocol) and 180 s-freeze-120 s-thaw-180 s freeze (180 s protocol). The study was divided into two parts. First, to evaluate embryo survival, fertilized chicken eggs were incubated. On embryonic day (ED) 12, cryoablation on CAM was performed according to the two protocols. During cryoablation, the temperature of the CAM was recorded using a thermal camera. Embryo survival was monitored until ED 14. Second, to evaluate tumor cryoablation, human neuroendocrine tumor cells (BON-1) were xenografted onto the CAM of fertilized chicken eggs at ED 8. Cryoablation of the xenografted tumors was then performed on ED 12 according to the two protocols. Ablation outcomes were evaluated by stereomicroscopic and histological assessments after harvesting on ED 14.

**Results:**

Embryo survival rates were 8/9 in both protocols. A decrease in the peripheral temperature of 4.5 (± 0.9) °C and 6.7 (± 1.0) °C was observed in the 120 s and 180 s protocols, respectively. Complete ablation of CAM-grown tumors was observed in 2/6 (120 s protocol) and 2/5 (180 s protocol) cases, few scattered tumor cells remaining in 2/6 (120 s protocol) and 2/5 (180 s protocol) cases. Residual interconnected tumor cells were visible in 2/6 (120 s protocol) and 1/5 (180 s protocol) cases.

**Conclusion:**

The CAM model is a feasible platform for preclinical cryoablation studies.

**Relevance statement:**

Chorioallantoic membrane model is a suitable platform for preclinical cryoablation research.

**Key Points:**

- Chick embryos tolerate the temperature drop during cryoablation well with high survival.
- Effectiveness of cryoablation on xenografted tumors can be histologically evaluated.
- Cryoablation protocols for xenografted tumors can be further optimized.

**Graphical Abstract:**

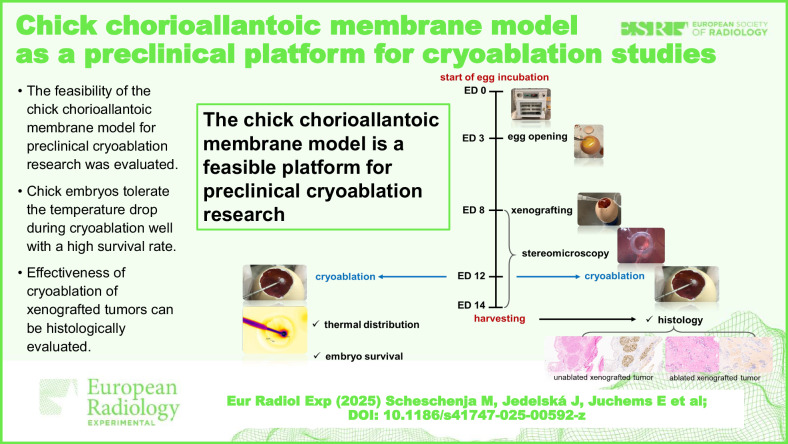

## Background

Percutaneous cryoablation is a minimally invasive technique that uses extreme cold to destroy tissues, including tumors. It is used for treatment of tumors in various organs such as kidney, prostate and lung as well as bone and soft tissue [1]. Advantages of cryoablation include its precision in targeting tumor tissue while preserving healthy tissue, its minimally invasive nature and the ability to perform the procedure without the need for extensive surgery. In selected cases, the procedure can even be performed under local anesthesia [1, 2].

In addition to the increasing use of cryoablation alone, the combination of ablation techniques with chemotherapy or even immunotherapy also appears to be promising [3–6]. Comprehensive preclinical research is essential to expand the range of applications, refine existing therapeutic strategies and evaluate combination therapies. Preclinical models can help to investigate cryoablation in a wider range of tumor types and evaluate its efficacy in combination with other therapies to identify optimal protocols and synergistic treatment combinations.

Traditional *in vitro* experiments are often unable to accurately mimic the complex interactions in a living organism. Although animal models are more representative, they are also associated with ethical concerns and high costs. Therefore, there is a need for alternative preclinical research methods that avoid the use of susceptible animals.

The chick chorioallantoic membrane (CAM) model is a promising alternative. The CAM is a highly vascularized extraembryonic membrane facilitating respiratory gas exchange and waste excretion during chick development [7]. As a preclinical research model, it has been employed to investigate various areas of pharmacological research [7, 8]. Moreover, tumor cells can be xenografted onto the CAM to form tumors that can be monitored in a biologically functional environment, making it a suitable and widely used model for tumor research [7, 9–11]. The CAM model offers benefits such as high reproducibility, cost-effectiveness, and ethical acceptability, as it uses non-sentient embryos [8]. In the future, it could allow investigation of tumor ablation in a biologically functional preclinical environment while reducing the use of sentient animals in preclinical research. Its feasibility has already been shown for different ablation techniques such as electroporation and radiofrequency ablation [12, 13].

The purpose of this study was to evaluate the CAM model for cryoablation procedures. This investigation aimed to determine whether the technique could be reliably performed on the CAM, whether chick embryos remained viable after the procedure, and whether the effects of ablation on xenografted tumors could be assessed histologically.

## Methods

All experiments in this prospective preclinical study were performed in compliance with the EU Directive 2010/63/EU on animal experiments. The Directive applies only to fetal forms of mammals from the last third of their normal development and does not classify avian embryos prior to hatching as protected animals [14]. Beyond that, all assays were terminated by embryonic day 14, prior to full nervous system maturation when pain, suffering or distress are not anticipated [15]. Therefore, these procedures are not considered animal experiments under the Directive and do not require ethics committee approval.

### Study design

The present study was divided into two sections: (1) the evaluation of chick embryo survival following cryoablation, and (2) the assessment of cryoablation on CAM-grown tumors. The methodology for egg incubation, preparation and xenografting was adapted from the established protocols for xenografting by Schulze et al and radiofrequency ablation as described by Wessendorf et al [11, 13].

### Egg incubation and preparation

Fertilized chicken eggs (Brormann GmbH) were allowed to equilibrate at room temperature for at least 2 h before incubation. Subsequently, the eggs were cleaned with 70% ethanol and incubated at 37.8 °C with 60–70% relative humidity in a hatching incubator (Brutmaschine Easy 150, J. Hemel Brutgeräte GmbH). On embryonic day (ED) 3, the eggs were removed from the incubator and cleaned again with 70% ethanol. Using a manual egg opener, scalpel and tweezers, a 2.5 cm window was carefully cut into the eggshell at the broad pole. Viable embryos were identified by clear blood vessels and a beating heart. The windows were then covered with sterile petri dishes, and the eggs were returned to the incubator in an upright position until further use.

### Cryoablation procedure

**Fig. 1:**
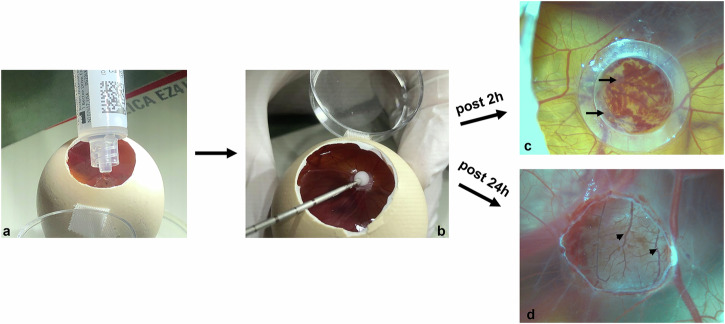
Cryoablation procedure on the chorioallantoic membrane (CAM) model. **a** Prior to ablation, a saline droplet was placed on the CAM. **b** The cryoprobe was manually positioned at an approximate 30–40° angle within the droplet, making contact with CAM. Cryoablation was initiated, resulting in the formation of a visible ice ball surrounding the tip of the cryoprobe. **c** Two hours after the procedure, hemorrhage (black arrows) was observed. Inner diameter of the silicone ring is 4 mm. **d** Clear evidence of intravascular coagulation (arrow heads) 24 h after cryoablation. The area subjected to cryoablation was localized within the ice ball. This became apparent following the removal of the silicone ring

All cryoablation procedures in both experimental parts were performed on embryonic day (ED) 12 using the Cryohit system (Galil Medical) with an ICESeed 1.5 cryoprobe. To ensure optimal thermal contact and homogeneous temperature distribution, a saline droplet was applied to the target area on the CAM before probe insertion (Fig. 1a). The cryoprobe was then manually positioned into the saline droplet on the CAM at an angle of approximately 30–40° relative to the CAM surface. In the cases of tumor cryoablation, the saline droplet was applied directly onto the tumor, and the cryoprobe was applied directly onto the main tumor mass (Fig. 1b). Two cryoablation protocols were established for the study. The first protocol consisted of a 120 s-120 s-120 s freeze-thaw-freeze sequence (120 s protocol), while the second protocol followed a 180 s-120 s-180 s freeze-thaw-freeze sequence (180 s protocol).

### Survival of the chick embryos

**Fig. 2:**
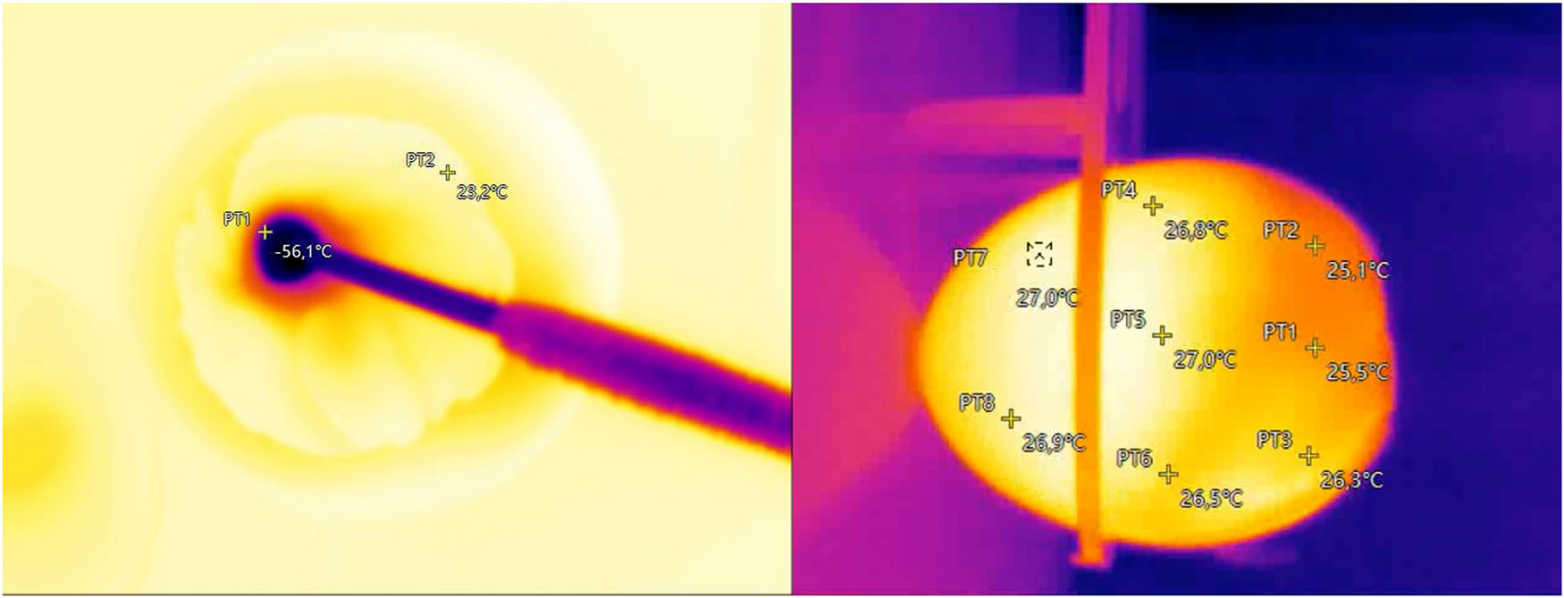
Thermal imaging of cryoablation on the chorioallantoic membrane (CAM) model. The left panel shows a top-down view of the egg with the CAM and tumor area, where the cryoprobe is applied at a 30–40° angle. A significant cooling effect is observed around the tip of the cryoprobe, indicating the localized impact of cryoablation. The right panel presents a side view of a different egg immediately after cryoablation, displaying a gradual temperature gradient from the pointed end of the egg toward the opening where the CAM is exposed, thus demonstrating the distribution of cooling throughout the egg after the procedure

As part of the first experiment, nine incubated eggs were designated to each protocol group for cryoablation. During the cryoablation process, temperatures were monitored using a thermal imaging camera (DytSpectrumOwl CA-20, Dianyang Technology Co., Ltd.). The temperature distribution and changes during the procedure were observed at two points: the ablation site and a peripheral point on the CAM (Fig. 2, left panel). Temperatures at the peripheral point were recorded directly before cryoablation and then at the end of the first freeze cycle, the thaw cycle and the last freeze cycle. Moreover, a side view of the temperature distribution during the cryoablation was recorded for three representative eggs (Fig. 2, right panel). The survival of the embryos was assessed at four time points: immediately post-cryoablation; 2 h post-cryoablation; 15 h post-cryoablation; and 40 h post-cryoablation.

### Cryoablation on xenografted tumors

As part of the second experiment, 4 days prior to cryoablation, human tumor cells (neuroendocrine tumor, BON-1) were xenografted on the CAM surface.

The cell line used for xenografting was the BON-1 (CVCL_3985) cell line, which was derived from a lymph node metastasis of a serotonin-producing neuroendocrine tumor of pancreas [16] and cultured in DMEM/HAM’s F12 medium + 10% FCS in a humidified atmosphere containing 5% CO_2_ at 37 °C. On ED 8, xenografting was performed by gently injuring the CAM with an aseptic lens tissue, followed by the placement of a silicone ring over the injured area. BON-1 cells (0.6 × 10^6^ in 20 µL medium) were mixed with 20 µL of Matrigel (Matrigel Matrix Basement Membrane, Corning, Bedford, USA) and implanted into the silicone ring. The eggs were then returned to the incubator, and tumor growth was monitored daily via stereomicroscopy. A total of 17 eggs were successfully xenografted and incubated. The CAM-grown tumors had macroscopically visible interconnected tumor tissue in all 17 eggs, typically with a thickness of approximately 1–2.5 mm.

The cryoablation was performed at ED 12 as described in the section “Cryoablation procedure”, in accordance with the two established cryoablation protocols. Six eggs were allocated to the 120 s protocol, five eggs were allocated to the 180 s protocol, and six eggs served as the sham cohort.

### Evaluation of outcomes

The survival of the embryos and stereomicroscopic changes were observed at the following time points: immediately after cryoablation, 2 h post-ablation, 15 h post-ablation, and 40 h post-ablation. On ED 14, CAM-tumors were harvested, fixed in phosphate-buffered 4% paraformaldehyde, and embedded in paraffin. Serial 3-µm paraffin sections were stained with hematoxylin-eosin (HE) for routine histological examination. Immunohistochemistry staining was performed using monoclonal mouse anti-human Ki67 antibodies (Dako, Ki67, Clone MIB-1) to mark proliferating BON 1 cells.

Stereomicroscopic observations and histologic evaluations of both HE- and immunohistochemistry-stained sections were conducted using a Leica EZ4 HD stereomicroscope and a Leica DM1000 LED microscope. The histologic changes and efficacy of tumor ablation were evaluated in both the HE- and immunohistochemistry-stained sections based on the extent of tumor cell destruction/necrosis as well as absence of Ki67 staining.

## Results

### Survival of the chick embryos

The cryoablation procedure was technically successful. Two hours post-procedure, hemorrhage was observed (Fig. 1c). However, after 24 h, there was clear evidence of intravascular coagulation resulting from the cryoablation procedure (Fig. 1d).

In the 120 s protocol group, only one embryo did not survive, resulting in a survival rate of 8 out of 9. Similarly, in the 180 s protocol group, one embryo did not survive either, yielding the same survival rate of 8 out of 9.

### Temperature distribution

In the 120 s protocol group, the mean temperature at the peripheral measuring point on the CAM before ablation was 29.6 °C (± 1.1), and the mean post-ablation temperature was 25.1 °C (± 1.6), resulting in an average temperature decrease of 4.5 °C (± 0.9). In the 180 s protocol group, the mean temperature at the peripheral measuring point on the CAM before ablation was 27.8 °C (± 0.9), and the mean post-ablation temperature was 21.1 °C (± 1.4), with an average temperature decrease of 6.7 °C (± 1.0). The temperature data are summarized in Table 1.

**Table 1:** Mean temperatures measured at various intervals throughout the experiment

Ablation protocol	Temperature (°C)					Survival rate
	Before ablation	End of the first freeze cycle	End of the thaw cycle	End of the second freeze cycle	Difference before and after cryoablation	
120 s protocol	29.6 ± 1.1	26.9 ± 1.3	26.9 ± 1.4	25.1 ± 1.6	4.5 ± 0.9	8/9 eggs
180 s protocol	27.8 ± 0.9	24.6 ± 1.1	24.2 ± 0.9	21.1 ± 1.4	6.7 ± 1.0	8/9 eggs

Temperature, given as mean ± standard deviation, was measured at a peripheral point on the CAM directly before ablation, then at the end of the first freeze cycle, at the end of the thaw cycle, and at the end of the second freeze cycle for both 120 s and 180 s protocol

### Cryoablation on xenografted tumors

**Fig. 3:**
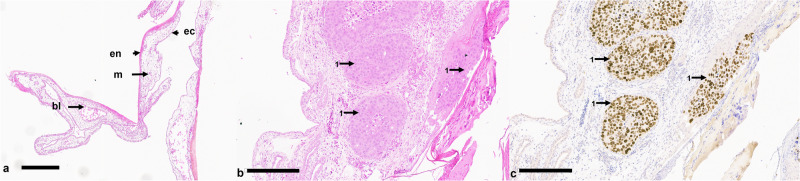
Histological section of a chorioallantoic membrane (CAM) and CAM-grown tumor that was not subjected to cryoablation. **a** Hematoxylin-eosin (HE)-stained CAM, which consists of ectoderm (ec), mesoderm (m) and endoderm (en). Blood vessels (bl) containing erythrocytes are formed in the mesoderm. **b** HE-stained section, highlighting cohesive tumor cell conglomerates (1). **c** Ki-67 staining, indicating numerous proliferating tumor cells (1). Note that the antibody is specific for human Ki-67; therefore, the cells in the CAM, although proliferating, are negative for Ki-67. Scale bars indicate 200 µm

The sham cohort exhibited six out of six survival rate with tumor growth in all six embryos, as confirmed both macroscopically and histologically (Fig. 3). The survival rates of the chick embryos were comparable in both ablation groups. In all cases, the embryos survived the cryoablation.

**Fig. 4:**
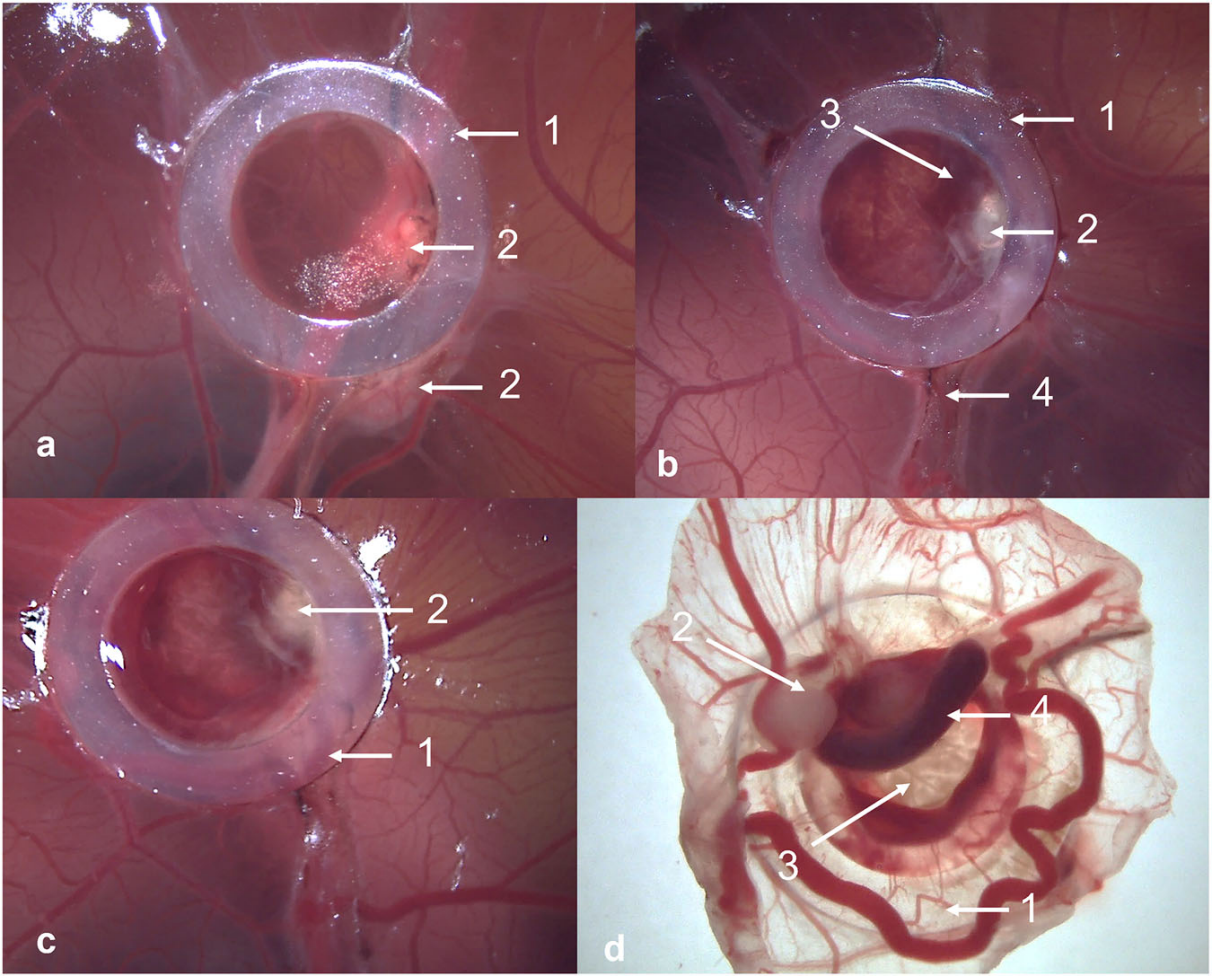
Stereomicroscopic images of the chorioallantoic membrane (CAM) and tumor progression before and post cryoablation. **a** Preablation image showing a vital tumor (2) at the edge of the silicone ring (1). **b** Image taken 24 h after cryoablation, displaying a nonvital, gray tumor (2), with signs of hemorrhage (3) and thrombosis (4). **c** Tumor in the CAM 48 h after ablation, exhibiting similar signs of tissue damage. **d** Excised tumor, viewed from the reverse side, showing a nonvital tumor (2) and pronounced evidence of thrombosis (4). Inner diameter of the silicone ring is 4 mm

**Fig. 5:**
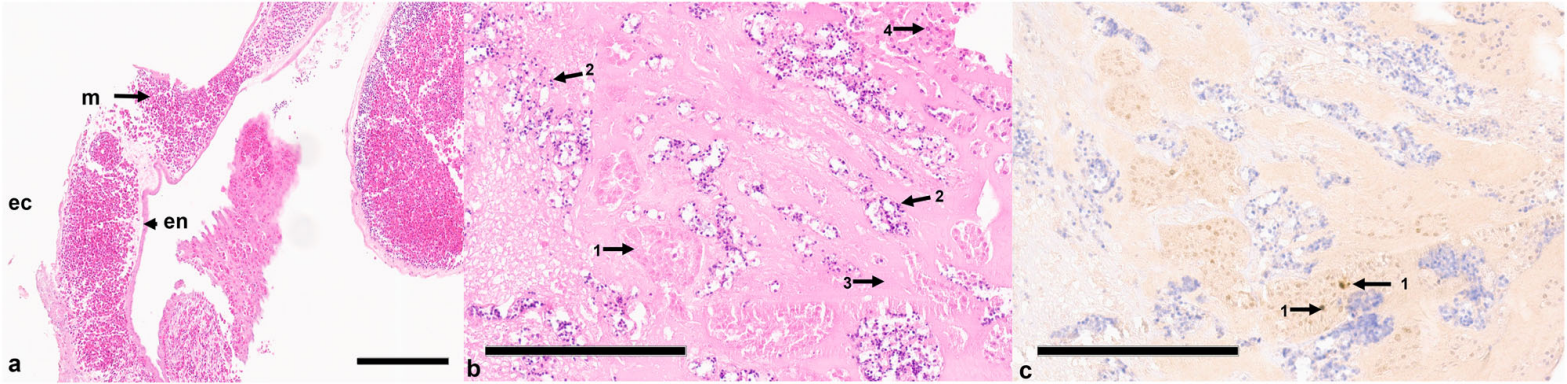
Histological section showing typical features following cryoablation. **a** Hematoxylin-eosin (HE)-stained cryoablated chorioallantoic membrane (CAM). The presence of significant hemorrhage, leukocyte infiltration and destruction of the ectoderm is clearly visible. **b** HE-stained section highlighting predominantly shrunken, necrotic tumor cells (1), leukocytes (2) remnants of Matrigel (3) and granulocytes (4). **c** individual, noncontiguous isolated vital tumor cells with positive Ki-67 staining, indicating residual proliferative activity despite ablation. Scale bars indicate 200 µm

Stereomicroscopic (Fig. 4) and histological (Fig. 5) findings in the cryoablation groups included tissue damage, bleeding, intravascular thrombosis, and leukocytic invasion. These observations were consistent across both the 120 s and 180 s protocol groups.

**Fig. 6:**
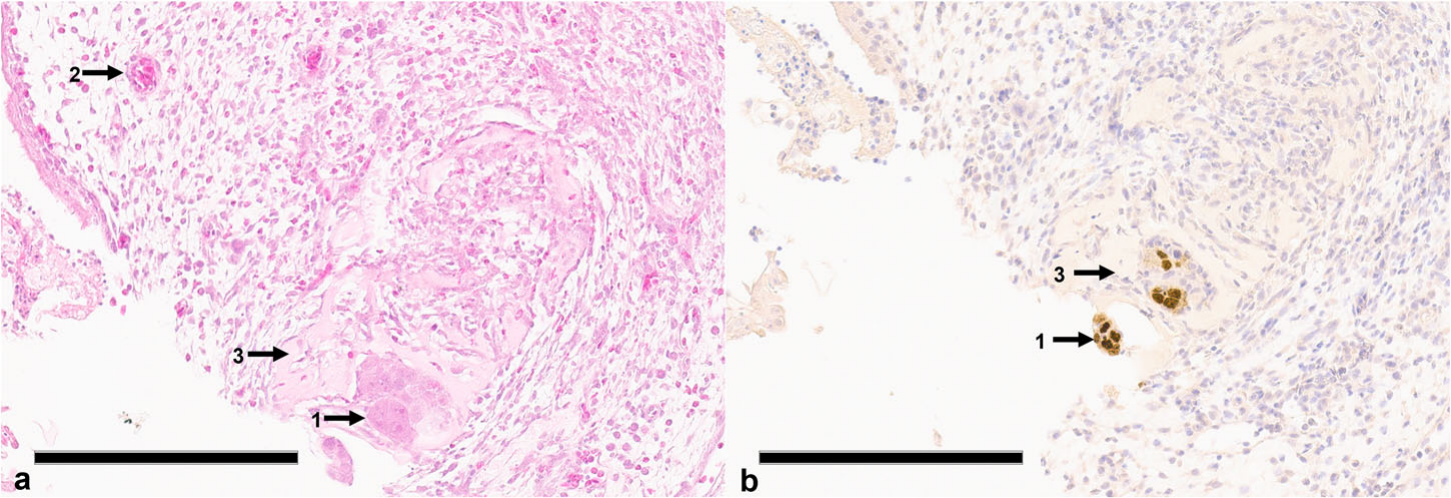
Histological section of chorioallantoic membrane (CAM)-grown tumor following cryoablation with incomplete results. **a** Hematoxylin-eosin (HE)-stained section highlighting cohesive tumor cell clusters (1) and a blood vessel filled with erythrocytes (2). **b** Ki-67 staining, indicating numerous proliferating tumor cells (1). Remnants of Matrigel can also be observed within the tumor tissue (3). Scale bars indicate 200 µm

In the 120 s protocol group, complete tumor ablation was observed in 2 out of 6 eggs. In 2 eggs, only a few scattered vital tumor cells remained, and in 2 eggs, an interconnected tumor conglomerate was observed. In the 180 s protocol group, complete tumor ablation was observed in 2 out of 5 eggs. In 2 eggs, only a few scattered vital tumor cells were remaining, and in 1 egg, an interconnected tumor conglomerate was observed. Exemplary histological sections from a complete and incomplete ablation are shown in Figs. 5 and 6.

## Discussion

Robust preclinical models are crucial for the development of innovative therapeutic approaches especially in the field of cancer research. In this context, the CAM model can build a bridge between *in vitro* experiments and ethically more and more discredited animal experiments. This study aimed to evaluate the feasibility of the use of the CAM model for preclinical research on cryoablation procedures.

Initially, our results demonstrated that the chick embryos tolerated the cryoablation procedure well, with a high survival rate observed in both experimental groups with different freezing durations. Although there was a temperature decrease in the periphery of the CAM in both groups during the cryoablation, this decrease, attributable to the surrounding room temperature and the cryoablation process itself, was well-tolerated by the embryos. The thermal imaging data indicated that the primary cooling effect was localized around the cryoprobe tip, suggesting a concentrated area of temperature drop. The overall decrease of temperature in the periphery of the CAM was measured to be 4.5 ± 0.9 °C and 6.7 ± 1.0 °C, respectively. The side-view thermal images revealed that the cooling effect penetrated the depth of the CAM but was not excessively deep. While these side-view images do not precisely reflect the internal conditions within the eggshell, they still can be seen as an indicator of temperature distribution. The observed temperature drop in the peripheral regions surrounding the ablation site was relatively low, suggesting limited cryogenic spread beyond the immediate vicinity of the cryoprobe. Chick embryos tolerate cold temperatures well. In previous studies, cold exposure has been used to immobilize embryos, particularly in preparation for MRI examinations, where embryos are cooled to 4 °C for up to 90 min without adverse effects [17, 18]. In comparison, the cold exposure during cryoablation, conducted at room temperature, is relatively mild and well-tolerated by the embryos. Thus, the CAM model appears to be a suitable and effective model for studying cryoablation. It is even conceivable that the embryos could withstand significantly longer and more intense freezing cycles.

In the second part of the experiment, our investigation was expanded to include CAM-grown tumors. Human neuroendocrine tumors (BON-1) were successfully xenografted onto the CAM, showing robust and consistent growth. The tumors in the sham cohort, where no cryoablation was performed, were well developed and easily detectable macroscopically and histologically, further supporting the suitability of the CAM as a reliable platform for tumor growth studies.

Cryoablation procedures resulted in both complete and partial tumor ablations. The observed stereomicroscopic and histological changes, including necrosis, bleeding, intravascular thrombosis, and leukocytic invasion, were consistent with the expected outcomes of cryoablation described in previous studies [19, 20].

Complete ablation was achieved in a third to 40% of the treated eggs, subtotal ablation was achieved in a further third to 40%. There were, however, 20% and 33% of remaining tumor cell conglomerates, pointing to a potential for further optimization.

One potential refinement could involve adjusting the freezing and thawing cycles, either by prolonging the freeze durations or applying repeated freeze-thaw cycles, which may improve ablation efficacy. Visible tumor conglomerates in the histological sections suggest that the cryoprobe may not have been adequately positioned on the tumor, resulting in incomplete coverage by the ice ball. Improving cryoprobe placement or utilizing different cryoprobes may also help reduce the occurrence of residual tumor tissue after ablation. Another approach to optimize the procedure could involve maintaining the eggs at a higher ambient temperature during cryoablation or applying methods to modify the efficacy of freezing and thawing. This adjustment could potentially reduce the stress on the embryos, thereby improving their overall survival rates and increasing their resilience to more aggressive ablation protocols or even combination therapies. Although incomplete ablations initially seem to represent a limitation, they also underscore a significant clinical challenge, as the presence of scattered residual vital tumor cells remains a persistent problem in cryoablation. However, this challenge opens up new research possibilities. The presence of residual tumor tissue after cryoablation in the CAM model offers an opportunity to explore combination therapies. For instance, combining cryoablation with adjunct treatments such as chemotherapy or immunotherapy could potentially improve therapeutic outcomes, particularly in cases where the tumor was not adequately covered by cryoablation.

The results of this study are consistent with previous research that demonstrated the viability of the CAM model for other ablation techniques. The successful application of radiofrequency ablation on the CAM model has been shown in previous studies. The use of the same tumor cell line in this research also underscores the feasibility of xenografting of the tumor cells on the CAM [13]. In addition, the model’s suitability for investigating electroporation has also been confirmed in earlier studies [12, 21]. The previous research, together with the results of the cryoablation, suggests that the CAM model is a valuable tool for preclinical research on ablation techniques, using a non-sentient, cost-effective, and reproducible platform.

The CAM model enables rapid and high-throughput testing, which is particularly advantageous for evaluating novel therapeutic strategies. Furthermore, the CAM model’s advantages over traditional animal models are significant. Traditional models, such as rodent or porcine systems, involve higher costs, complex logistical requirements and ethical concerns. In contrast, experiments on the CAM model, relying on non-sentient chick embryos, adhere to the principles of the Three Rs (Replacement, Reduction, and Refinement), minimizing animal welfare concerns and ethical issues associated with other animal models, making it an attractive alternative for preclinical studies. Its suitability for the preclinical evaluation of cryoablation across various tumor types is particularly noteworthy, as the model’s biological environment supports the investigation of the diverse mechanisms underlying cryoablation, including direct cellular damage and indirect vascular-mediated injury [22, 23].

Several limitations must be acknowledged. First, the use of chick embryos as an experimental model is inherently limited by species differences. Avian embryos are not mammals; therefore, their tissue responses and overall physiology may not fully replicate those observed in mammalian systems. Moreover, the immune system of chick embryos is still in development, which could lead to altered tumor–host interactions. Another limitation is the relatively short time interval between xenografting and harvest. To remain compliant with ethical and animal welfare guidelines, the experimental period was necessarily brief, allowing for only a limited observation window immediately after cryoablation. This may result in an underestimation of delayed cell death or longer-term tumor regression, as residual tumor cells observed shortly after the procedure might have undergone complete ablation at later time points. Finally, technical challenges such as optimal cryoprobe placement and ensuring complete coverage of the target tissue may have contributed to incomplete ablation in some cases, underscoring the need for further optimization.

In conclusion, the CAM model offers a promising and versatile platform for preclinical studies on tumor cryoablation. Its ability to support robust tumor growth, coupled with the high tolerance of chick embryos to cryoablation-induced temperature changes, underscores its suitability for further research. The findings of this study pave the way for future investigations aimed at optimizing cryoablation techniques and exploring innovative therapeutic combinations, contributing to the advancement of cancer treatment strategies. It can be expected that the CAM model will find broader application for specific preclinical cancer research in the field of tumor ablation.

## Data Availability

The datasets used during the current study are available from the corresponding author on reasonable request.

## Abbreviations

CAM: Chorioallantoic membrane
ED: Embryonic day
HE: Hematoxylin-eosin

## References

[1] Cazzato RL, Garnon J, Ramamurthy N et al (2016) Percutaneous image-guided cryoablation: current applications and results in the oncologic field. Med Oncol 33:140. 10.1007/s12032-016-0848-3 27837451 10.1007/s12032-016-0848-3

[2] Tatli S, Acar M, Tuncali K et al (2010) Percutaneous cryoablation techniques and clinical applications. Diagn Interv Radiol 16:90–95. 10.4261/1305-3825.DIR.1922-08.0 19998248 10.4261/1305-3825.DIR.1922-08.0

[3] Aarts BM, Klompenhouwer EG, Rice SL et al (2019) Cryoablation and immunotherapy: an overview of evidence on its synergy. Insights Imaging 10:53. 10.1186/s13244-019-0727-5 31111237 10.1186/s13244-019-0727-5 PMC6527672

[4] Yakkala C, Denys A, Kandalaft L, Duran R (2020) Cryoablation and immunotherapy of cancer. Curr Opin Biotechnol 65:60–64. 10.1016/j.copbio.2020.01.006 32088576 10.1016/j.copbio.2020.01.006

[5] Baust JM, Santucci KL, Van Buskirk RG et al (2022) An in vitro investigation into cryoablation and adjunctive cryoablation/chemotherapy combination therapy for the treatment of pancreatic cancer using the PANC-1 cell line. Biomedicines 10:450. 10.3390/biomedicines10020450 35203660 10.3390/biomedicines10020450 PMC8962332

[6] Clarke DM, Baust JM, Van Buskirk RG, Baust JG (2004) Addition of anticancer agents enhances freezing-induced prostate cancer cell death: implications of mitochondrial involvement. Cryobiology 49:45–61. 10.1016/j.cryobiol.2004.05.003 15265716 10.1016/j.cryobiol.2004.05.003

[7] Ribatti D (2016) The chick embryo chorioallantoic membrane (CAM). A multifaceted experimental model. Mech Dev 141:70–77. 10.1016/j.mod.2016.05.003 27178379 10.1016/j.mod.2016.05.003

[8] Kundeková B, Máčajová M, Meta M et al (2021) Chorioallantoic membrane models of various avian species: differences and applications. Biology 10:301. 10.3390/biology10040301 33917385 10.3390/biology10040301 PMC8067367

[9] Ribatti D (2014) The chick embryo chorioallantoic membrane as a model for tumor biology. Exp Cell Res 328:314–324. 10.1016/j.yexcr.2014.06.010 24972385 10.1016/j.yexcr.2014.06.010

[10] Chu P-Y, Koh AP-F, Antony J, Huang RY-J (2021) Applications of the chick chorioallantoic membrane as an alternative model for cancer studies. Cells Tissues Organs 211:222–237. 10.1159/000513039 33780951 10.1159/000513039 PMC9153341

[11] Schulze J, Librizzi D, Bender L et al (2023) How to xenograft cancer cells on the chorioallantoic membrane of a fertilized hen’s egg and its visualization by PET/CT and MRI. ACS Appl Bio Mater 6:2435–2445. 10.1021/acsabm.3c00237 37222633 10.1021/acsabm.3c00237

[12] Fiorentzis M, Viestenz A, Siebolts U et al (2019) The potential use of electrochemotherapy in the treatment of uveal melanoma: in vitro results in 3D tumor cultures and in vivo results in a chick embryo model. Cancers (Basel) 11:1344. 10.3390/cancers11091344 31514412 10.3390/cancers11091344 PMC6769976

[13] Wessendorf J, Scheschenja M, Bastian M et al (2023) Feasibility of the chick chorioallantoic membrane model for preclinical studies on tumor radiofrequency ablation. Eur Radiol Exp 7:56. 10.1186/s41747-023-00368-3 37749303 10.1186/s41747-023-00368-3 PMC10519884

[14] Directive 2010/63/EU of the European parliament and of the council of 22 September 2010 on the protection of animals used for scientific purposes. Available online: https://eur-lex.europa.eu/eli/dir/2010/63/2019-06-26 (Accessed on 15 Sep 2024).

[15] Weiss L, Saller AM, Werner J et al (2023) Part I: Analysis of cardiovascular responses to a mechanical noxious stimulus. Animals (Basel) 13:2710. 10.3390/ani13172710 37684974 10.3390/ani13172710 PMC10486618

[16] Evers BM, Townsend CM, Upp JR et al (1991) Establishment and characterization of a human carcinoid in nude mice and effect of various agents on tumor growth. Gastroenterology 101:303–311. 10.1016/0016-5085(91)90004-5 1712329 10.1016/0016-5085(91)90004-5

[17] Herrmann A, Taylor A, Murray P et al (2018) Magnetic resonance imaging for characterization of a chick embryo model of cancer cell metastases. Mol Imaging 17:1536012118809585. 10.1177/1536012118809585 30392458 10.1177/1536012118809585 PMC6236852

[18] Zuo Z, Syrovets T, Genze F et al (2015) High-resolution MRI analysis of breast cancer xenograft on the chick chorioallantoic membrane. NMR Biomed 28:440–447. 10.1002/nbm.3270 25711154 10.1002/nbm.3270

[19] Bhardwaj N, Strickland AD, Ahmad F et al (2009) A comparative histological evaluation of the ablations produced by microwave, cryotherapy and radiofrequency in the liver. Pathology 41:168–172. 10.1080/00313020802579292 19152189 10.1080/00313020802579292

[20] Seifert JK, Gerharz CD, Mattes F et al (2003) A pig model of hepatic cryotherapy. In vivo temperature distribution during freezing and histopathological changes. Cryobiology 47:214–226. 10.1016/j.cryobiol.2003.10.007 14697733 10.1016/j.cryobiol.2003.10.007

[21] Tsimpaki T, Anastasova R, Liu H et al (2024) Calcium electroporation versus electrochemotherapy with bleomycin in an in vivo CAM-based uveal melanoma xenograft model. Int J Mol Sci 25:938. 10.3390/ijms25020938 38256012 10.3390/ijms25020938 PMC10815639

[22] Song KD (2016) Percutaneous cryoablation for hepatocellular carcinoma. Clin Mol Hepatol 22:509–515. 10.3350/cmh.2016.0079 28081593 10.3350/cmh.2016.0079 PMC5266346

[23] Erinjeri JP, Clark TWI (2010) Cryoablation: mechanism of action and devices. J Vasc Interv Radiol 21:S187–S191. 10.1016/j.jvir.2009.12.403 20656228 10.1016/j.jvir.2009.12.403 PMC6661161

